# Phase relationships in two-dimensional mass spectrometry

**DOI:** 10.1007/s13361-019-02308-1

**Published:** 2019-10-15

**Authors:** Maria A. van Agthoven, David P. A. Kilgour, Alice M. Lynch, Mark P. Barrow, Tomos E. Morgan, Christopher A. Wootton, Lionel Chiron, Marc-André Delsuc, Peter B. O’Connor

**Affiliations:** 1 Department of Chemistry, University of Warwick, Gibbet Hill Road, Coventry, CV4 7AL UK; 2 Present Address: School of Science and Technology, Nottingham Trent University, 50 Shakespeare Street, Nottingham, NG1 4FQ UK; 3 Present Address: Department of Computer Science, University of St Andrews, North Haugh, St Andrews, Fife, KY16 9SX UK; 4 CASC4DE, Le Lodge 20 av. du Neuhof, 67100 Strasbourg, France; 5 Institut de Génétique et de Biologie Moléculaire et Cellulaire, INSERM, U596, CNRS, UMR7104, Université de Strasbourg, 1 rue Laurent Fries, 67404 Illkirch-Graffenstaden, France

**Keywords:** Fourier transform, Mass spectrometry, Two-dimensional mass spectrometry, Phase correction, Data processing

## Abstract

Two-dimensional mass spectrometry (2D MS) is a data-independent tandem mass spectrometry technique in which precursor and fragment ion species can be correlated without the need for prior ion isolation. The behavior of phase in 2D Fourier transform mass spectrometry is investigated with respect to the calculation of phase-corrected absorption-mode 2D mass spectra. 2D MS datasets have a phase that is defined differently in each dimension. In both dimensions, the phase behavior of precursor and fragment ions is found to be different. The dependence of the phase for both precursor and fragment ion signals on various parameters (e.g., modulation frequency, shape of the fragmentation zone) is discussed. Experimental data confirms the theoretical calculations of the phase in each dimension. Understanding the phase relationships in a 2D mass spectrum is beneficial to the development of possible algorithms for phase correction, which may improve both the signal-to-noise ratio and the resolving power of peaks in 2D mass spectra.

**Electronic supplementary material:**

The online version of this article (10.1007/s13361-019-02308-1) contains supplementary material, which is available to authorized users.

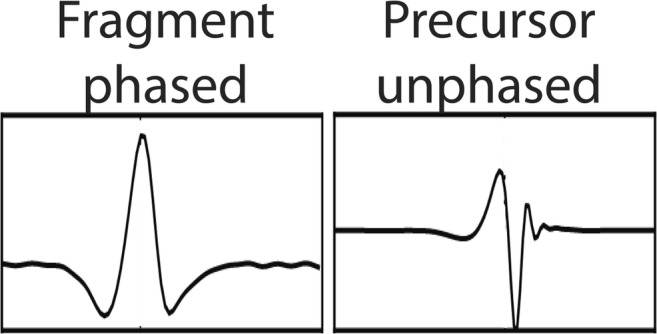

## Introduction

Two-dimensional mass spectrometry (2D MS) is a data-independent tandem mass spectrometry technique in which precursor and fragment ion species can be correlated without the need for prior ion isolation [1]. The fragmentation patterns of all ions from a complex sample can be mapped in a single 2D mass spectrum from one experiment. The experiment time and sample consumption are independent of sample complexity. Because there is no ion isolation and the ion signals are multiplexed, the signal-to-noise improves with the resolving power for more accurate precursor-fragment ion correlation. Using isotopic distributions of fragment ions, the correlation between precursor and fragment ions can be achieved through their *m/z* ratio and their charge state [2].

The first 2D MS pulse sequence was proposed by Pfändler et al. for a Fourier transform ion cyclotron resonance mass spectrometer (FT-ICR MS) in 1987 [3–7]. For two decades, the demands of 2D MS in terms of data storage and processing exceeded commonly available computational capacities. The theory of 2D MS, as well as alternative pulse sequences for ion radius modulation such as stored waveform ion radius modulation and Hadamard transformation, was proposed and studied [8–11]. In 2010, 2D MS was revived and successfully applied to a commercial FT-ICR mass spectrometer with infrared multiphoton dissociation (IRMPD), electron capture dissociation (ECD), and infrared activated electron capture dissociation (IR-ECD) as fragmentation methods [12–14]. Denoising algorithms have been proposed in order to decrease the amount of scintillation noise in 2D mass spectra and the pulse sequence was optimized [15–17]. 2D MS has been applied to small molecules and bottom-up and top-down proteomics, as well as polymer analysis [18–25]. Automated peak-assignment algorithms are being developed for routine analysis [26]. A pulse sequence for 2D MS in a linear ion trap has been proposed, which expands the potential of 2D MS to mass analyzers beyond FT-ICR MS [27–29]. Nevertheless, the question of how to improve the signal-to-noise ratio and the resolving power of 2D mass spectra remains. One significant improvement has been proposed with the use of a non-uniform sampling technique [30]. Another answer can be phase correction for absorption mode 2D mass spectra.

In one-dimensional FT-ICR mass spectrometry, phase correction for absorption mode mass spectra was solved by Xian et al. and Qi et al. [31, 32]*.* In FT-ICR MS experiments, ion packets start at the center of the ICR cell and require excitation to higher radii to be detectable. Ions are excited by a broadband radiofrequency (RF) voltage on the excitation plates of the ICR cell. Their motion starts accruing phase (i.e., the position in the ICR cell in polar coordinates) when they are in resonance with the RF voltage pulse. The phase of the ion packets in the ICR cell at the start of detection is determined by the moment ions start accruing phase, which depends on the ions’ cyclotron frequency and the broadband excitation pulse. The phase of each ion packet at the start of detection determines the phase of the signal they generate on the detection plates. The Fourier transform of the signal results in an absorption mode spectrum and a dispersion mode spectrum, which both contain amplitude and phase information for each cyclotron frequency. The mass spectrum plots amplitude as a function of cyclotron frequency. Most often, this information is obtained by calculating the magnitude mode spectrum, in which there is no phase information. Recovering the phase of the signal as a function of the cyclotron frequency (i.e., the phase function) enables the calculation of the signal amplitude as a function of cyclotron frequency, i.e., phase-corrected absorption-mode spectrum.

Phase-corrected, absorption-mode, one-dimensional mass spectra show improved signal-to-noise ratio (up to a factor of 1.41) and resolving power (up to a factor of 2) [33]. Automated phase correction for absorption mode mass spectra was achieved with the development of the Autophaser software by Kilgour et al. [34–36]. Phase correction for absorption mode mass spectrometry is now integral to data processing for FT-ICR MS, for various geometries of ICR cells [35, 37]. Phase-corrected absorption-mode data from the dynamically harmonized ICR cell has allowed the highest published FT-ICR MS resolving power (*R* = 32,000,000 at *m/z* 400) [37]. The effect of various instrument parameters on phase functions has also been studied [33]. Apodisation functions have been developed for absorption mode spectra, as well as peak shape distribution models [38, 39]. Absorption mode FT-ICR MS has shown particular promise for complex mixture analysis such as the study of proteins, crude oil, and lignin [40–43]. Phase correction for absorption mode FTMS has also been developed for Orbitraps and electrostatic linear ion traps [44, 45].

In this article, the behavior of phase in 2D mass spectra is studied. First, the different behavior of the phase of precursor and fragment ion peaks in each transient of the 2D data set is discussed. Then, the phase behavior for precursor and fragment ions is discussed as a function of the encoding delay *t*_1_, in particular how the phase signal behavior is informed by dissociation mechanisms. Experimental data is shown to be congruent with the theory for the phase behavior in each dimension. Finally, the use the phase information to improve the resolution and the signal-to-noise of a 2D mass spectrum is discussed.

## Experimental methods

### Sample preparation

Substance P and angiotensin 1 were purchased from Sigma-Aldrich (Dorset, UK) and used as received. Substance P was dissolved in a 1 pmol/μL solution of water/methanol/formic acid (49.95:49.95:0.1 vol). Angiotensin 1 was dissolved in a 1 pmol/μL solution of water/acetonitrile/formic acid (75:24.9:0.1 vol). The water was deionized using a Direct-Q 3 Ultrapure Water System (Millipore, Nottingham, UK). Methanol and acetonitrile were purchased from VWR International Ltd (Lutterworth, UK). Formic acid was purchased from Sigma-Aldrich (Dorset, UK).

### Instrument parameters

All experiments were performed on a 12 T solariX Fourier transform ion cyclotron resonance mass spectrometer (Bruker Daltonik, GmbH, Bremen, Germany). Substance P and angiotensin 1 were ionized using a home-built nanoelectrospray (nESI) ion source at a rate of approximately 7–8 μL/h. After transfer through a dual ion funnel, two octopoles and a quadrupole ions were accumulated in a hexapole-based collision cell for 0.1 s and transferred through a transfer hexapole (1.0-ms transfer period) to the infinity cell for fragmentation and detection [46].

**Scheme 1:**
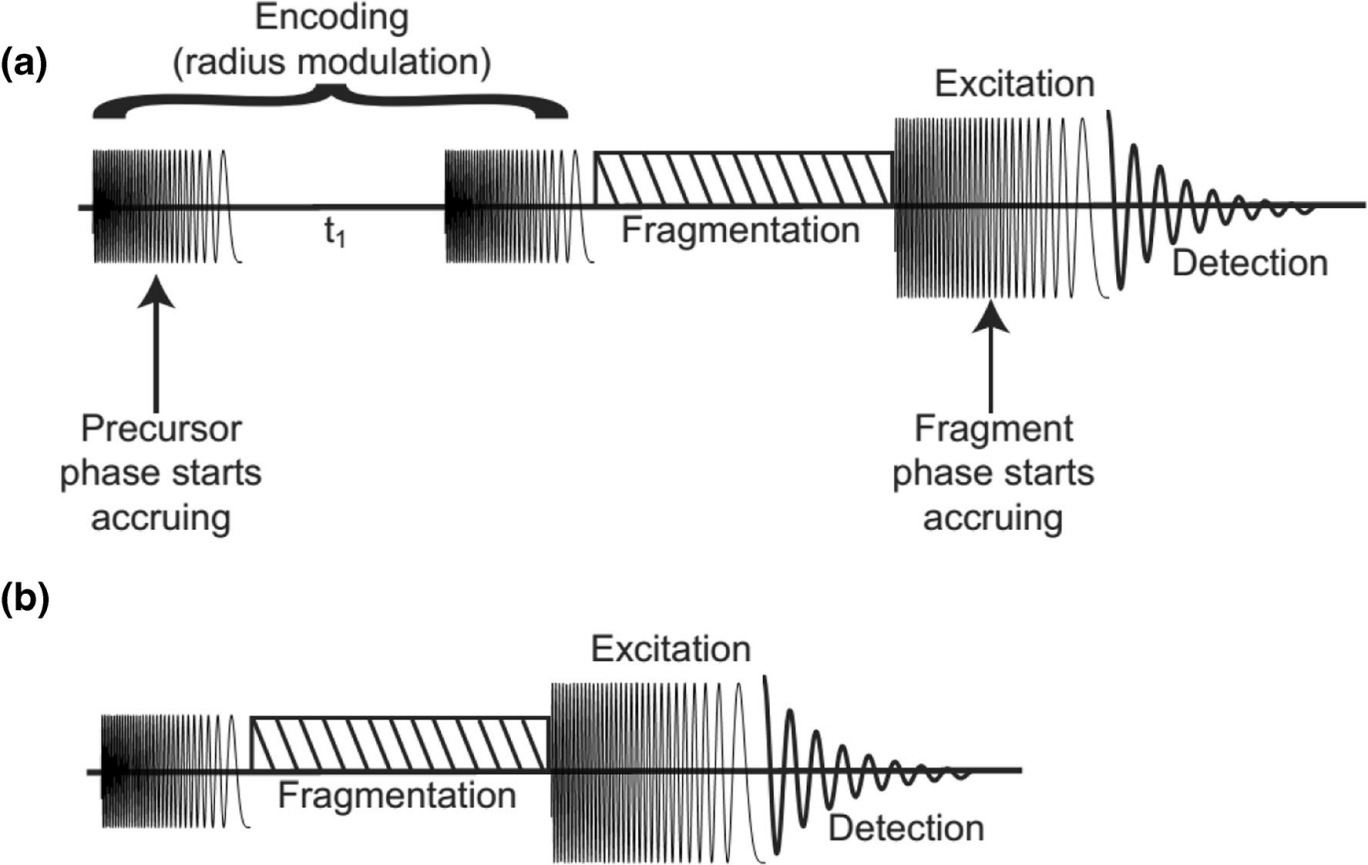
(**a**) Experimental script of Gäumann’s pulse sequence for 2D FT-ICR MS. (**b**) Experimental script of pulse sequence to map fragmentation zones and to optimize the parameters for the 2D MS pulse sequence

**Figure 1:**
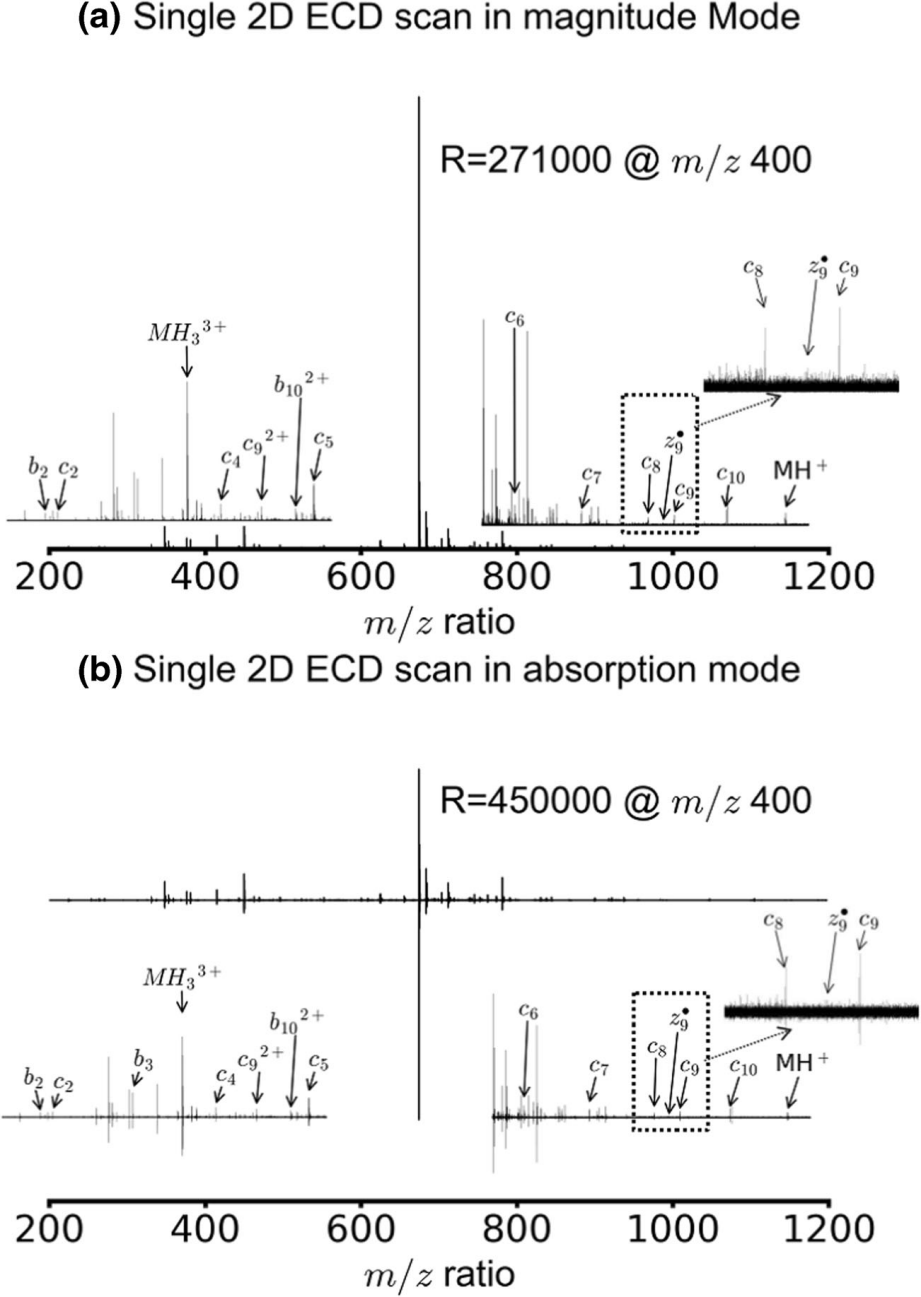
ECD tandem mass spectrum for substance P (MH_3_^3+^ and MH_2_^2+^ precursor ions) using the pulse sequence in Scheme 1a with a *t*_1_ delay of 1.0 μs (**a**) in magnitude mode (**b**) in absorption mode. The insets on the left are zoom-ins of the spectra between *m/z* 200–650 and the insets on the right are zoom-ins of the spectra between *m/z* 680–1400

**Figure 2:**
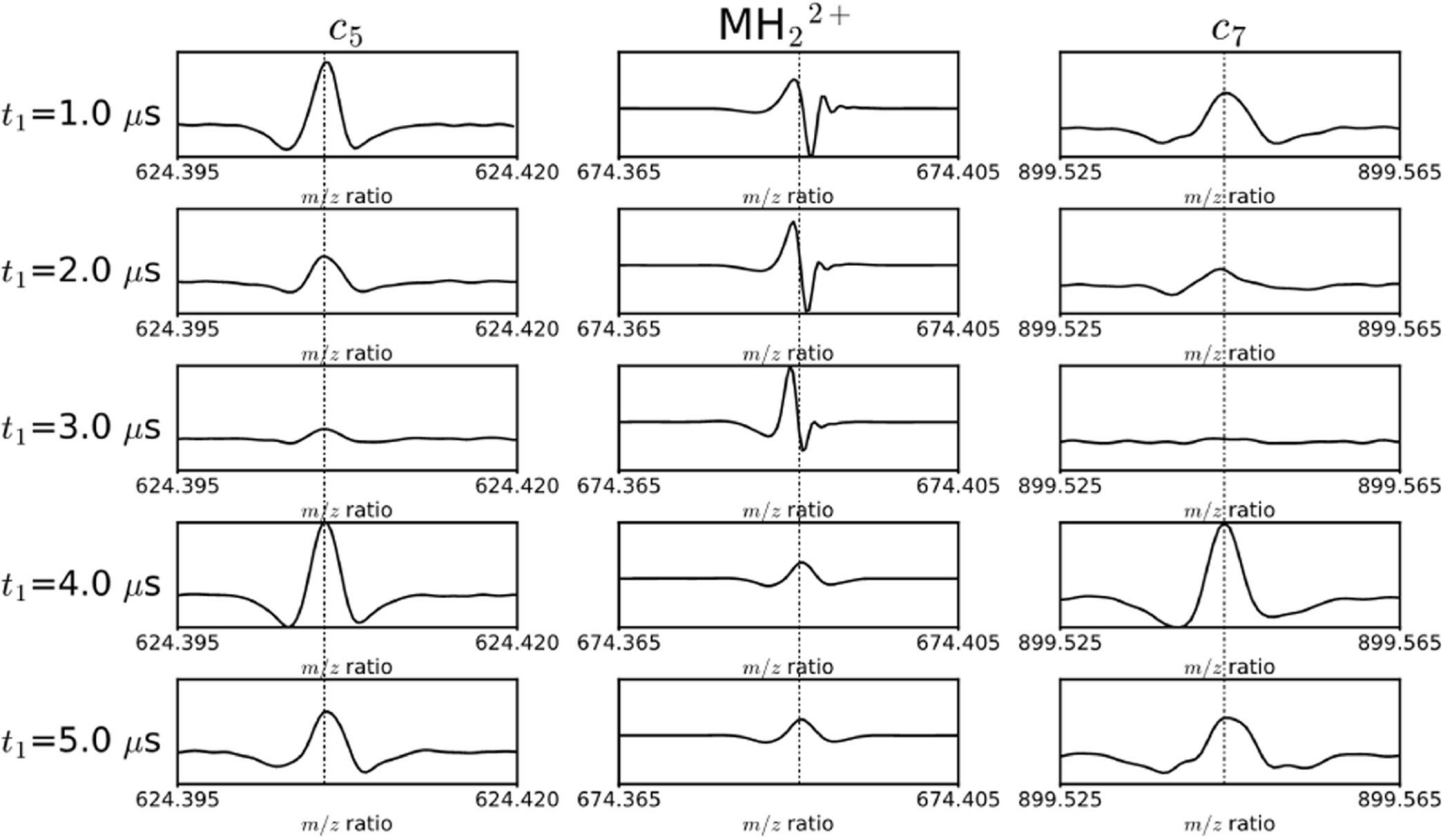
Absorption mode ECD tandem mass spectra for substance P using the pulse sequence in Scheme 1a with a *t*_1_ delay of 1.0–5.0 μs (*t*_1_ delay of 1.0 is from the same spectrum as shown in Figure 1b). The column in the middle shows the peak assigned to the MH_2_^2+^ precursor. The columns on the left and right show the peak for the *c*_5_ and *c*_7_ fragments for different values of *t*_1_ respectively

ECD tandem mass spectra were measured for substance P with the pulse sequence shown in Scheme 1a (data shown in Figures 1 and 2). The single scan transients were recorded with 4 Mwords (16 bits) over 1.6777 s for a mass range of *m/z* 147.4–1500 (1250-–122.8-kHz frequency range). All three pulses had a 70 *V*_pp_ amplitude. The first two pulses had 4 μs per frequency in 1804 decrements of 625 Hz. The third pulse had 20 μs per frequency in 1804 decrements of 625 Hz. The values of the delay between the two first pulses were 1.0 μs, 2.0 μs, 3.0 μs, 4.0 μs, and 5.0 μs. ECD was performed using electrons from a 1.5-A indirectly heated hollow cathode dispenser [47]. The ECD lens was set at 60 V, the ECD bias at 1.8 V, and the irradiation period was 0.1 s.

**Figure 3:**
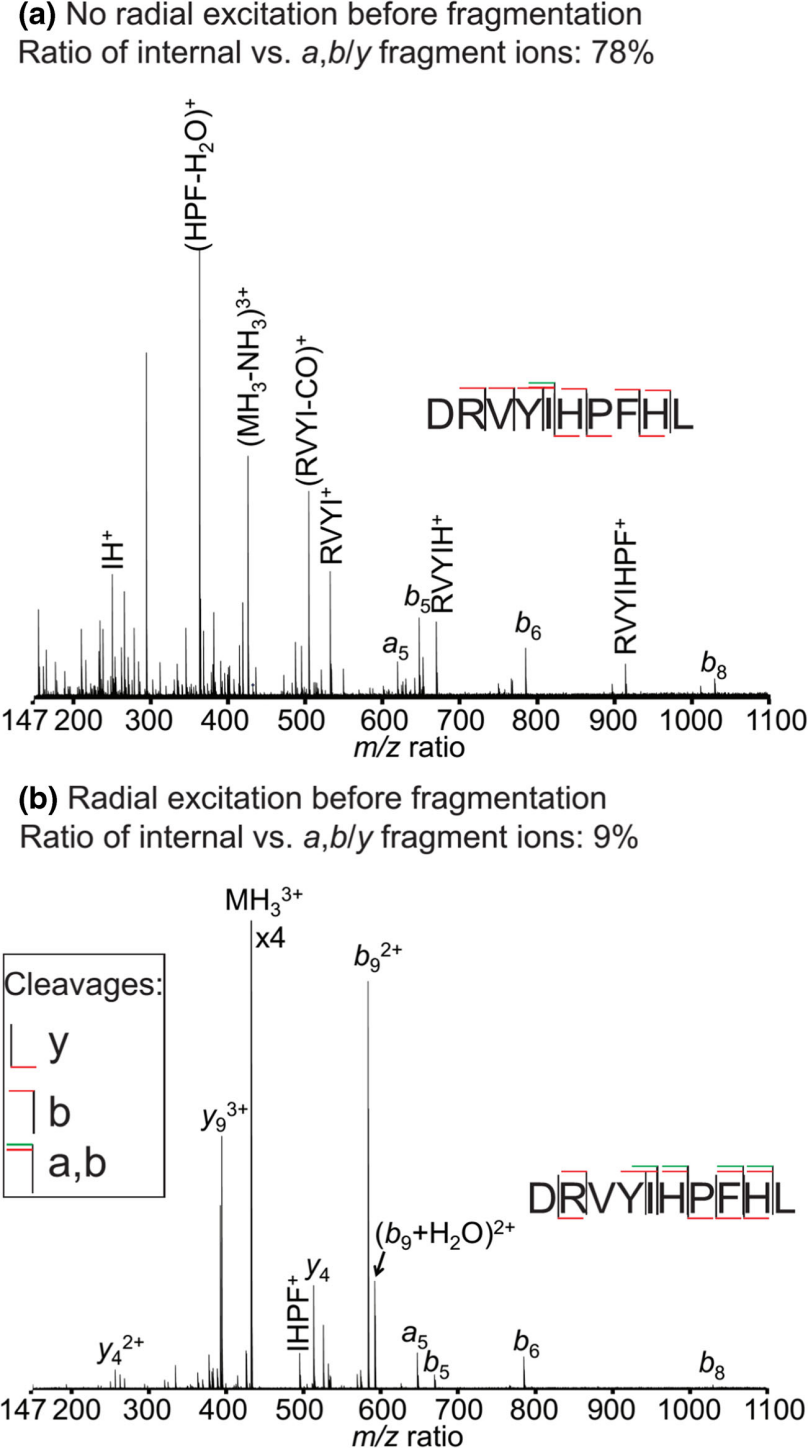
Magnitude mode IRMPD tandem mass spectra for angiotensin 1 using the pulse sequence in Scheme 1b with an amplitude of (**a**) 1 *V*_pp_ and (**b**) 31 *V*_pp_ for the first pulse of the pulse sequence

**Figure 4:**
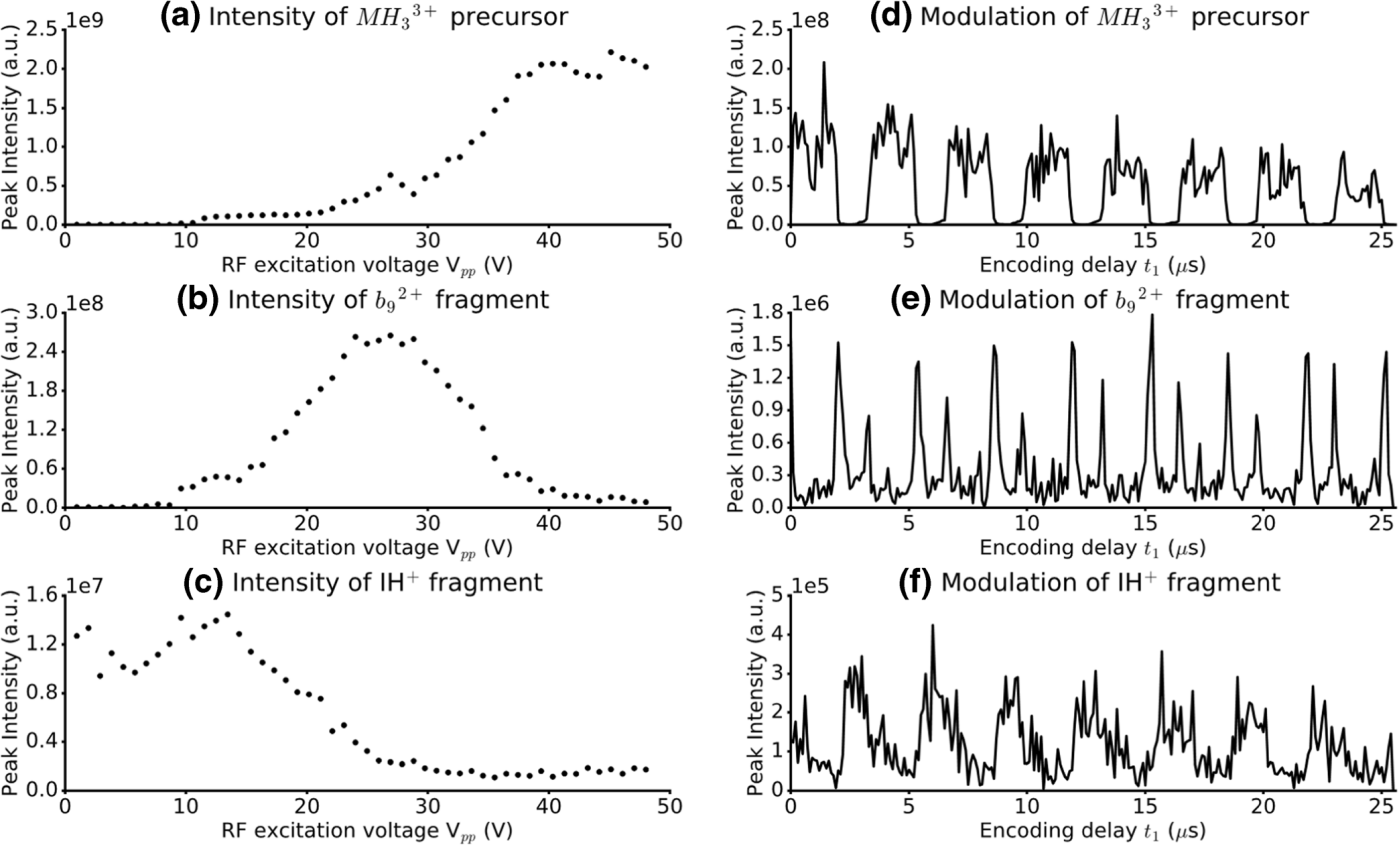
Left side: Ion intensity vs. RF excitation voltage for the pulse sequence in Scheme 1b for (**a**) MH_3_^3+^ of angiotensin, (**b**) the *b*_9_^2+^ fragment, and (**c**) the IH^+^ internal fragment. Right side: Ion intensity vs. encoding delay *t*_1_ for the pulse sequence in Scheme 1a for (**d**) MH_3_^3+^ of angiotensin, (**e**) the *b*_9_^2+^ fragment, and (**f**) the IH^+^ internal fragment

IRMPD tandem mass spectra were measured for angiotensin 1 with the pulse sequence shown in Scheme 1b (data shown in Figures 3 and 4a–c). The precursor ion at *m/z* 433 was isolated in the front-end quadrupole. The transients were recorded over 20 scans with 4 Mwords (16 bits) over 1.6777 s for a mass range of *m/z* 147.4–1500 (1250-–122.8-kHz frequency range). The first pulse had 10 μs per frequency in 1804 decrements of 625 Hz and an amplitude varying from 0 to 50 *V*_pp_. The second pulse had an amplitude of 70 *V*_pp_ with 20 μs per frequency in 1804 decrements of 625 Hz. IRMPD was performed using a Synrad 48-2 CO_2_ 25-W laser (Mukilteo, WA, USA) with a 10.6-μm wavelength at 50% power with a 0.55-s irradiation period.

**Figure 5:**
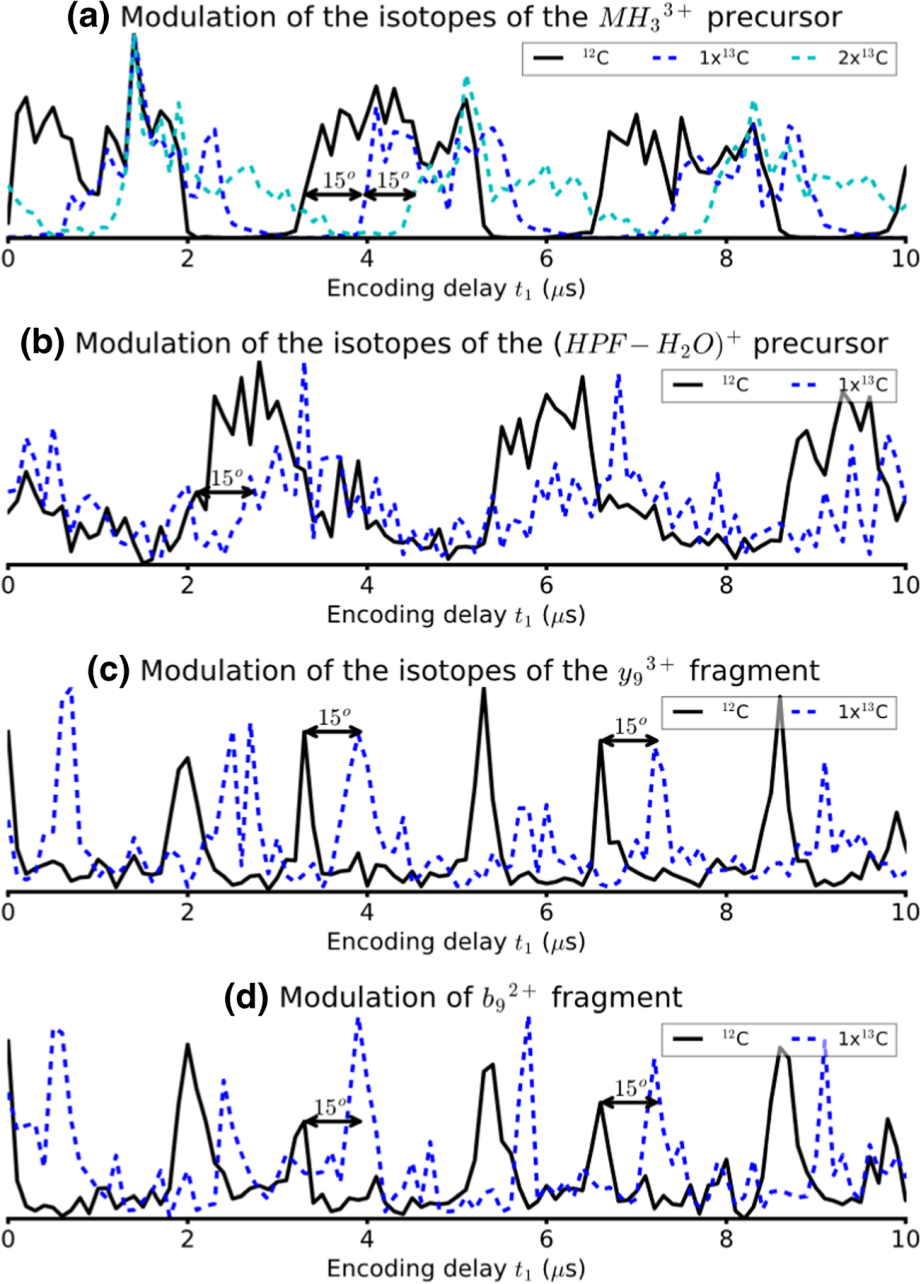
Normalized ion intensity vs. encoding delay *t*_1_ for the pulse sequence in Scheme 1a for (**a**) the ^12^C, 1×^13^C, and 2×^13^C isotopes of MH_3_^3+^ of angiotensin 1, (**b**) the ^12^C and 1×^13^C isotopes of the (HPF-H_2_O)^+^ fragment at *m/z* 364.1768, (**c**) the ^12^C and 1×^13^C isotopes of the *y*_9_^3+^ fragment at *m/z* 394.5575, and (**d**) the ^12^C and 1×^13^C isotopes of the *b*_9_^2+^ fragment at *m/z* 583.2987

IRMPD tandem mass spectra were measured for angiotensin 1 with the pulse sequence shown in Scheme 1a (data shown in Figures 4d–f and 5). The precursor ion at *m/z* 433 was isolated in the front-end quadrupole. The single scan transients were recorded with 4 Mwords (16 bits) over 1.6777 s for a mass range of *m/z* 147.4–1500 (1250-–122.8-kHz frequency range). The first two pulses had an amplitude of 70 *V*_pp_ with 0.4 μs per frequency in 1804 decrements of 625 Hz. The delay *t*_1_ was incremented 256 times from 0.1 to 25.6 μs. The third pulse had an amplitude of 70 *V*_pp_ with 20 μs per frequency in 1804 decrements of 625 Hz. IRMPD was performed using a Synrad 48-2 CO_2_ 25-W laser (Mukilteo, WA, USA) with a 10.6-μm wavelength at 50% power with a 0.55-s irradiation period.

### Data processing

The mass spectra were visualized in the DataAnalysis 4.0 software (Bruker Daltonik, GmbH, Bremen, Germany). All spectra were externally calibrated with the solariXcontrol software (Bruker Daltonics, Billerica, MA, USA) using Agilent ESI-L Low Concentration Tuning Mix (Agilent Technologies, Stockport, UK). Internal calibration was achieved using a quadratic calibration equation in the Data Analysis 4.0 software (Bruker Daltonik, GmbH, Bremen, Germany) [48, 49].

The absorption mode spectra of the ECD tandem mass spectra of substance P were obtained by using a quadratic phase function that was optimized with the Autophaser software with mass exclusions with *m/z* 1 window around *m/z* 353.3, *m/z* 449.9, and *m/z* 781.1 after asymmetric apodisation (*F* = 0.25) [34, 35, 38, 39].

The IRMPD mass spectra of angiotensin 1 with the pulse sequence from Scheme 1a were zerofilled, apodized, and Fourier transformed according to their acquisition rate in the SPIKE software developed independently by the University of Strasbourg and CASC4DE (Illkirch-Graffenstaden, France) in 64-bit Python programming language on a commercial platform distributed by Anaconda Continuum Analytics (Austin, TX, USA) [50].

## Theory

The pulse sequence for 2D MS in an FT-ICR mass spectrometer is shown in Scheme 1a. In the first pulse, all precursor ions are coherently excited to a radius that is dependent solely on the amplitude and length of the excitation pulse [51]. After the first pulse, the ions packets are left to orbit in the ICR cell at their own reduced cyclotron frequency for a period *t*_1_. The second pulse is identical to the first one. The interaction between the pulse and an ion packet depends on the position of the ion packet when resonant excitation starts. When the ion packet is in phase with the excitation voltage (i.e., the potentials on the excitation plates are attractive when the ion packet is closest to them), it is excited to a higher radius. When the ion packet is in the opposite phase to the excitation voltage (i.e., the potentials on the excitation plates are repelling when the ion packet is closest to them), it is de-excited to a lower radius. The final radius of the precursor ion packet *r*(*t*_1_) can be expressed as follows:

1 $$ r\left({t}_1\right)={r}_0{\left(2\left(1+\cos {\omega}_1\left({t}_1+T\right)\right)\right)}^{1/2} $$

in which *r*_0_ is the radius of the precursor ion packet at the end of the first pulse, *ω*_1_ is the reduced cyclotron frequency of the precursor ions, *T* is the duration of each pulse, and *t*_1_ is the period between the two first pulses [8]. As a result, the radius of the precursor ions is encoded with their reduced cyclotron frequency.

After the second pulse, a radius-dependent fragmentation period (using ECD, IRMPD, IR-ECD, or ion-neutral collisions, for example) leads to fragment ion abundances that depend on the value of *t*_1_ and the reduced cyclotron frequency of their precursor ions. The third pulse is applied in order to excite all ions to higher radius before detection. The pulse sequence is repeated multiple times with regularly incremented values of *t*_1_ to modulate the precursor ion packet radii in and out of the central fragmentation zone in the ICR cell prior to irradiation with an infrared laser (IRMPD) or reaction with electrons (ECD). In contrast with the ion-neutral collision method initially proposed, ECD and IRMPD are applied to ions located near the center of the cell, and in any case, ions rotating with a radius negligible compared with the final radius accessed after the final excitation pulse will interact with the laser or electron beam and fragment [3, 4, 14, 17].

A scan is acquired and recorded for each value of *t*_1_ to produce the dataset. A signal *s*(*t*_1_, *t*_2_) is measured as a function of *t*_1_ (delay between the two pulses) and *t*_2_ (transient). The signal presents two different phase evolutions. For each transient, the ion motion in the ICR cell leads to different phases for each frequency [31, 32]. For each value of *t*_1_, the amplitude of the signal is modulated [8]. In multidimensional nuclear magnetic resonance (NMR) spectroscopy, two-dimensional signals with a *t*_1_ amplitude modulation have been shown to require processing using hypercomplex Fourier transformation [52–55]. Since the signals in 2D MS also have a *t*_1_ amplitude modulation, data processing is also accomplished through hypercomplex Fourier transformation [56]. The spectrum is obtained by calculating the hypercomplex Fourier transform according to *t*_2_ and *t*_1_:

2 $$ S\left({\omega}_1,{\omega}_2\right)={\int}_{t_1=-\infty}^{+\infty }{\int}_{t_2=-\infty}^{+\infty }s\left({t}_1,{t}_2\right){e}^{j{\omega}_2{t}_2}{e}^{i{\omega}_1{t}_1}d{t}_2d{t}_1 $$

in which *S* is the signal in the frequency space and *ω*_1_/2π and *ω*_2_/2π are the frequencies, and *i* and *j* are constants:

3 $$ {i}^2={j}^2=-1 $$

The resulting spectrum is a 4 quadrant data set:

4 $$ S\left({\omega}_1,{\omega}_2\right)= RR\left({\omega}_1,{\omega}_2\right)+ iRI\left({\omega}_1,{\omega}_2\right)+ jIR\left({\omega}_1,{\omega}_2\right)+ kII\left({\omega}_1,{\omega}_2\right) $$

The hypercomplex generators *i*, *j*, and *k* follow these rules:

5 $$ i\cdotp j=j\cdotp i=k\ \mathrm{and}\ {k}^2=1 $$

6 $$ i\cdotp k=k\cdotp i=-j $$

7 $$ j\cdotp k=k\cdotp j=-i $$

The magnitude mode spectrum *F*(*ω*_1_, *ω*_2_) can be obtained using the hypercomplex modulus for each data point and results in the loss of any phase information:

8 $$ F\left({\omega}_1,{\omega}_2\right)={\left( RR{\left({\omega}_1,{\omega}_2\right)}^2+ RI{\left({\omega}_1,{\omega}_2\right)}^2+ IR{\left({\omega}_1,{\omega}_2\right)}^2+ II{\left({\omega}_1,{\omega}_2\right)}^2\right)}^{1/2} $$

Phase corrections can also be applied independently to *S*(*ω*_1_, *ω*_2_) along the *ω*_1_ and *ω*_2_ axes:

9 $$ S\left({\omega}_1,{\omega}_2\right){e}^{i{\varphi}_1}=S\left({\omega}_1,{\omega}_2\right)\left(\mathit{\cos}{\varphi}_1+ isin{\varphi}_1\right) $$

10 $$ S\left({\omega}_1,{\omega}_2\right){e}^{j{\varphi}_2}=S\left({\omega}_1,{\omega}_2\right)\left(\mathit{\cos}{\varphi}_2+ jsin{\varphi}_2\right) $$

As Eqs. (9) and (10) show, the phase in the horizontal dimension and the phase in the vertical dimension are independent from each other. Until now, only the magnitude mode spectrum *F*(*ω*_1_, *ω*_2_) has been used in studies of 2D MS. For a phase-corrected absorption-mode 2D mass spectrum, phase correction can be applied either along the horizontal fragment ion dimension or along the vertical precursor ion dimension, and knowing how the phase of the signal evolves with frequency is necessary. The phase contributions of the ion motion in each transient and from the ion intensities are therefore studied for the pulse sequence proposed by Pfändler et al. [3, 4]*.*

### Phases of precursor ions in the horizontal fragment ion dimension

Scheme 1a shows the pulse sequence that was developed by Pfändler et al. and which has been used in most 2D MS studies [3, 4]. Precursor ions are first coherently excited during the first pulse, which is a frequency sweep [57]. Xian et al. [58] and Qi et al. [32] have shown that the phase accrued by ion packets at the end of a frequency sweep can be expressed as:

11 $$ {\varphi}_{\mathrm{a}}={c}_2{\omega_1}^2+{c}_1{\omega}_1+{c}_0 $$

in which *φ*_a_ is the phase accrued at the end of the first pulse, *ω*_1_/2π is the cyclotron frequency of the ion packet, and *c*_0_, *c*_1_, and *c*_2_ are constants that are dependent on the parameters of the frequency sweep.

During the encoding delay *t*_1_, each coherent precursor ion packet rotates at its own cyclotron frequency and accrues a phase *φ*_b_ that can be expressed as:

12 $$ {\varphi}_{\mathrm{b}}={\omega}_1{t}_1 $$

During the second encoding pulse, the coherent precursor ion packet continues to accrue phase continuously despite the change in cyclotron radius that is dependent on the delay *t*_1_ (vide infra) [4]. The phase accrued is:

13 $$ {\varphi}_{\mathrm{c}}={\omega}_1{T}_{\mathrm{pulse}} $$

in which *φ*_c_ is the phase accrued during the second pulse and *T*_pulse_ is the duration of the pulse. Some precursor ion packets, depending on their cyclotron frequency and the value of *t*_1_, can go back to the center of the ICR cell at the end of the second pulse, in which case their phase is not defined anymore (in polar coordinates, the phase is undefined at the origin). Precursor ions that are at the center of the ICR cell at the end of the second pulse and which are not fragmented are only coherently excited again during the third excitation pulse. The behavior of their phase is therefore the same as the behavior of the phase of fragment ions.

Precursor ion packets that are not at the center of the ICR cell at the end of the second pulse and that are not fragmented during the fragmentation period continue to accrue phase:

14 $$ {\varphi}_{\mathrm{d}}={\omega}_1{T}_{\mathrm{fragmentation}} $$

in which *φ*_d_ is the phase accrued during fragmentation and *T*_fragmentation_ is the duration of the fragmentation period.

Finally, precursor ion packets also continue to accrue phase during the excitation pulse:

15 $$ {\varphi}_{\mathrm{e}}={\omega}_1{T}_{\mathrm{e}\mathrm{xcitation}} $$

in which *φ*_e_ is the phase accrued during excitation and *T*_excitation_ is the duration of the excitation pulse.

As a result, the phase *φ*_p_(*ω*_1_) that has been accrued by precursor ion packets at the beginning of detection, which determines the phase of the peak at *ω*_1_ in the frequency spectrum, can be expressed as:

16 $$ {\varphi}_{\mathrm{p}}\left({\omega}_1\right)={\varphi}_{\mathrm{a}}+{\varphi}_{\mathrm{b}}+{\varphi}_{\mathrm{c}}+{\varphi}_{\mathrm{d}}+{\varphi}_{\mathrm{e}} $$

17 $$ {\varphi}_{\mathrm{p}}\left({\omega}_1\right)={c}_2{\omega_1}^2+{c}_1{\omega}_1+{c}_0+{t}_1{\omega}_1+{T}_{\mathrm{p}\mathrm{ulse}}{\omega}_1+{T}_{\mathrm{fragmentation}}{\omega}_1+{T}_{\mathrm{excitation}}{\omega}_1 $$

18 $$ {\varphi}_{\mathrm{p}}\left({\omega}_1\right)={c}_2{\omega_1}^2+{c_1}^{\prime }{\omega}_1+{c}_0+{t}_1{\omega}_1 $$

in which

19 $$ {c_1}^{\prime }={c}_1+{T}_{\mathrm{pulse}}+{T}_{\mathrm{fragmentation}}+{T}_{\mathrm{excitation}} $$

As Eqs. (18) and (19) show, *c*_2_, *c*_1_*′*, and *c*_0_ do not depend on *t*_1_, but the phase function of precursor ions is dependent on *t*_1_ and precursor ion packets have a different phase function for each transient that is recorded in the 2D MS experiment. This phase function only applies if ions are not de-excited back to the ICR cell after the second pulse.

### Phases of fragment ions in the horizontal fragment ion dimension

Because all known fragmentation methods have efficiencies that are independent of the phase of the precursor ion’s position in the ICR cell, fragment ions can be generated at any phase. In addition, except for ultraviolet photodissociation, fragmentation periods are typically several orders of magnitude longer than the cyclotron periods of precursor ions [59]. Fragment ion packets at the end of the fragmentation period are always centered in the ICR cell and can be considered non-coherent, regardless of the size and shape of the ion packet. Therefore, fragment ion packets (as well as surviving precursor ion packets at the center of the ICR cell) start accruing phase during the excitation pulse, in which they are coherently excited. The sweep rate of the excitation pulse, for an optimized pulse sequence [17], is often different from the sweep rate of the encoding pulses. The phase of the fragment ion packet at the start of the detection can therefore be expressed as:

20 $$ {\varphi}_{\mathrm{f}}\left({\omega}_2\right)={c_2}^{\prime \prime }{\omega_2}^2+{c_1}^{\prime \prime }{\omega}_2+{c}_0^{\prime\prime } $$

in which *φ*_f_(*ω*_2_) is the phase of the fragment ion packets at the start of detection, *ω*_2_ is the reduced cyclotron frequency of the fragment, and *c*_2_*″*, *c*_1_*″*, and *c*_0_*″* are constants that depend on the sweep rate and the parameters of the experiment.

Equation (20) shows that fragment ion packets have a phase function that is independent of the encoding delay *t*_1_, which is the only change to the pulse sequence from transient to transient in the 2D mass spectrometry experiment. The comparison between Eqs. (18), (19), and (20) shows that precursor and fragment ions have different phase values. In addition, sometimes the same ion *m/z* can be a precursor present at the start of the pulse sequence and a fragment generated from another precursor during the fragmentation period. Therefore, two ion packets with the same cyclotron frequency but with different phases (e.g., an ion species that is both a precursor and a fragment of another ion species) can coexist during detection.

Qi et al. have studied the experimental parameters that can affect the phase function [33]. In a 2D MS experiment, only the delay *t*_1_, the total ion number, and the excitation radius change from scan to scan in the experiment. As Qi et al. show, the phase function is stable over large variations in total ion numbers and excitation radii [33]. As a result, factors *c*_2_, *c*_1_*′*, *c*_0_, *c*_2_*″*, *c*_1_*″*, and *c*_0_*″* are expected to show little variation from scan to scan.

### Phases of precursor and fragment ions in the vertical precursor ion dimension

In the vertical precursor ion dimension, the spectrum is obtained by calculating the Fourier transform of the ion intensities for each cyclotron frequency according to the encoding delay *t*_1_. Guan and Jones [8] have shown that the radius modulation of the precursor ion packets *r*(*t*_1_) can be expressed as:

$$ r\left({t}_1\right)={r}_0{\left(2\left(1+\cos {\omega}_1\left({t}_1+T\right)\right)\right)}^{1/2} $$

in which *r*_0_ is the radius of the precursor ion packet at the end of the first pulse, *ω*_1_ the reduced cyclotron frequency of the precursor ions, *T* the duration of each pulse, and *t*_1_ the period between the two first pulses [8]. After the second pulse, the radius of the precursor ion packet is modulated between 0 and 2*r*_0_, with *r*(*t*_1_ = 0) = *r*_0_(2(1 + cos *ω*_1_*T*))^1/2^.

For fragment ions, phase behavior is dependent on the fragmentation method. In IRMPD, the fragmentation efficiency can be considered to have a Gaussian distribution with the radius (depending on laser beam profile) if there are no secondary fragmentations [17]. As a result, the abundance of fragment ions at the end of the fragmentation period can be expressed as:

21 $$ \alpha \left({t}_1\right)={\alpha}_0{e}^{-a{\left(\raisebox{1ex}{$r\left({t}_1\right)$}\!\left/ \!\raisebox{-1ex}{${R}_{\mathrm{laser}}$}\right.\right)}^2} $$

in which *α*(*t*_1_) is the abundance of the fragments generated at *t*_1_, *α*_0_ is the abundance of the fragment ions generated at the center of the ICR cell, *a* is a normalization factor, *r*(*t*_1_) is the radius of the precursor ion packet at *t*_1_, and *R*_laser_ is the radius of the laser beam in the ICR cell. Equations (1) and (21) can be combined:

22 $$ \alpha \left({t}_1\right)={\alpha}_0{e}^{-2a\left(1+\mathit{\cos}{\omega}_1{t}_1\right)\times {\left({r}_0/{R}_{\mathrm{laser}}\right)}^2} $$

For *t*_1_ = 0, *α*(*t*_1_) is at its minimum. For values of *t*_1_ that are odd multiples of π/*ω*_1_, *α*(*t*_1_) is at its maximum. In IRMPD, the intensity of fragment ion peaks as a function of *t*_1_ (vertical dimension) is therefore out of phase by π or 180° with the intensity of precursor ion peaks.

For collisionally activated dissociation (CAD), fragmentation efficiency is proportional to the kinetic energy of the precursor ion and therefore increases with the radius of the precursor ion packet:

23 $$ \alpha \left({t}_1\right)\propto \frac{1}{2} qB\omega {r}_0\left(1+\mathit{\cos}{\omega}_1{t}_1\right) $$

in which *α*(*t*_1_) is the abundance of the fragments generated at *t*_1_, *B* is the magnetic field, *q* is the charge of the precursor ion, and *r*_0_ is the radius of the precursor ion packet at the end of the first pulse. For CAD, fragment ion intensity as a function of *t*_1_ is in phase with the radius modulation of the precursor ions.

IRMPD and CAD show two extremes in terms of phase difference between precursor ion peaks and fragment ion peaks. Fragmentation zones can have more complicated distributions, e.g., in the case of hollow cathode ECD, secondary fragmentations, or dual fragmentation methods like IR-ECD, in which both an IR laser and an electron cathode are involved [14, 47]. In these cases, the phase difference between the precursor and the fragment ion intensities as a function of *t*_1_ can vary. As a result, the contribution of the vertical dimension to the phase information of each peak depends on the fragmentation method, the fragmentation parameters, and the nature of the precursor ion and the fragment ion.

## Results and discussion

### Phases of ions in the horizontal fragment ion dimension

Figure 1 shows the ECD scan of substance P obtained with the pulse sequence shown in Scheme 1a with *t*_1_ = 1.0 μs. No quadrupole isolation was applied before the 2D MS pulse sequence. Figure 1 a shows the spectrum in magnitude mode. Two precursor ions can be assigned in the spectrum MH_3_^3+^ and MH_2_^2+^ of substance P, as well as fragments that are characteristic of ECD (see peak assignments in Table S2 in the Online Resource). The resolving power was 271,000 FWHM at *m/z* 400.

Figure 1 b shows the same data set in phase-corrected absorption mode. The phase function used to correct the phase in the spectrum was calculated with mass exclusions that ensured that the phase function was calculated using only fragment ion peaks in the mass spectrum. The resolving power of the spectrum was 513,000 FWHM at *m/z* 400, which corresponds to a 1.9 factor improvement from the magnitude mode spectrum, which may be partly due to the fact that different apodisation functions were used in magnitude mode (sine-bell multiplication) and absorption mode (asymmetrical apodisation) [38, 60]. With the same apodisation functions, the improvement was found to be of a factor 1.7. For the precursor ions of substance P (MH_2_^2+^ and MH_3_^3+^), the phase correction is inaccurate and the peak shapes are distorted. This result is consistent with the theoretical calculations showing that the phase of the fragment ion peaks in a transient from a 2D MS data set is determined by the third pulse in the pulse sequence (Eq. (20)), but that the phase of the precursor ion peaks depends on the whole pulse sequence (Eq. (19)).

To demonstrate the phase dependences of the precursor and fragments on *t*_1_, Figure 2 shows the phase-corrected absorption mode for ECD tandem spectra of substance P that have been acquired using the 2D pulse sequence shown in Scheme 1a for different values of the delay *t*_1_. The middle column shows the evolution of the intensity of the MH_2_^2+^ precursor ion, the left column shows the evolution of the intensity of the *c*_5_ fragment, and the right column shows the evolution of the intensity of the *c*_7_ fragment. For each ion species, the intensity is shown on the same scale, the external calibration function is the same, and the phase function is the same for all values of *t*_1_. The phase function was calculated with the same mass exclusions for each spectrum, as in Figure 1b [34]. All five mass spectra were internally calibrated (the mass spectra before mass calibration are shown in Fig. S2 in the Online Resource).

Figure 2 shows that both the *c*_5_ and the *c*_7_ fragment peaks are properly phase-corrected for all values of *t*_1_. The peak for the MH_2_^2+^ precursor, however, is not phase-corrected for *t*_1_ values between 1.0 and 3.0 μs, but is properly phase-corrected for *t*_1_ values of 4.0 and 5.0 μs. The phase accrued by the MH_2_^2+^ precursor (at a frequency of 273 kHz) during the delay *t*_1_ in these spectra is 16°, 31°, 47°, 63°, and 79°. The value of *t*_1_ therefore significantly affects the phase of the precursor ion peak, but does not affect the phase of the fragment ion peaks. The phases of precursor ion peaks are dependent on *t*_1_, and the phases of fragment ion peaks are independent of *t*_1_.

In Figure 2, the intensities of the *c*_5_ and *c*_7_ fragments decrease between *t*_1_ = 1.0 and 3.0 μs and both reach a minimum at *t*_1_ = 3.0 μs. The intensities increase again for *t*_1_ = 4.0 and 5.0 μs. The intensities of the *c*_5_ and *c*_7_ fragments are synchronized with respect to *t*_1_ because the two fragments have the same fragmentation efficiency profile, which is consistent with ECD cleaving inter-residue bonds non-preferentially for substance P (in Fig. S3 of the Online Resource, all five spectra were phase-corrected with the same phase coefficients that were optimized in Autophaser using the dataset in Fig. S1: the precursor and fragment peaks show the same behavior as in Figure 2) [61].

### Phases of ions in the vertical precursor ion dimension

Figure 3 shows two MS/MS spectra of MH_3_^3+^ of angiotensin 1 with the pulse sequence shown in Scheme 1b and after ion isolation using the front-end quadrupole of the mass spectrometer. The pulse sequence in Scheme 1b is used to optimize the parameters of the 2D MS pulse sequence [14, 17]. The ion packets are excited by the first pulse in the sequence to a radius that is determined by the amplitude and the length of the pulse [51]. After a fragmentation period, all precursor and fragment ions are excited by the second pulse and detected. Here, the fragmentation method is IRMPD, which has an approximately Gaussian-shaped fragmentation zone [17].

In Figure 3a, the amplitude of the first pulse is 1 *V*_pp_ with a pulse length of 10 μs per frequency, which corresponds to 0.7% radial excitation compared with the default excitation pulse in the Bruker solariXcontrol software (70 *V*_pp_ and 20 μs per frequency). The fragmentation efficiency, as determined by the ratio between the intensity of all the fragment ion peaks and the intensity of fragment and precursor ion peaks, was calculated to be 99% from the spectrum. This result is consistent with the fact that the precursor ion packet overlaps with the region of maximum photon density of the IRMPD laser beam. Since fragment ion packets are also in the region of maximum photon density during the irradiation period, secondary fragmentation can be expected to occur. For IRMPD fragmentation of peptides, internal ions are generally accepted to be the product of secondary fragmentation of primary *a*,*b*/*y* fragments [62]. Among the assigned fragments in the spectrum in Figure 3a, the intensity ratio of internal fragment ions was 78% (see Table S3 in the Online Resource).

In Figure 3b, the amplitude of the first pulse was 31 *V*_pp_ with a pulse length of 10 μs per frequency, which corresponds to 22% radial excitation compared with the default excitation pulse. Because of the radial excitation, the precursor ion packet moves to a region of lower photon density than in Figure 3a, and the fragmentation efficiency was 52%. Secondary fragmentation can also be expected to be lower, and the intensity ratio of internal ions among assigned fragments was 9% (see Table S4 in the Online Resource).

The comparison between the spectra in Figure 3a and b shows that internal fragment ions (i.e., secondary fragments of MH_3_^3+^ of angiotensin 1) have a different radial fragmentation efficiency profile in the ICR cell to primary *a*,*b*/*y* fragments [14]. Therefore, the behavior of internal fragments in the 2D IRMPD MS experiment of peptides can be hypothesized to be different from the behavior of primary *a*,*b*/*y* fragments as a function of *t*_1_.

Another comparison between Figure 3a and b shows that the cleavage coverage of the peptide is incomplete for each spectrum, but that the combination of the two mass spectra with different photon densities leads to complete cleavage coverage of the angiotensin 1 peptide. Since in 2D MS experiments precursor ions fragment at different energies due to the radius modulation, the resulting 2D mass spectrum shows peaks for all the fragment ions generated within the fragmentation energy range, which may lead to improved cleavage coverage over standard MS/MS experiments where only one fragmentation energy level is used. In this aspect, 2D MS is similar to MS^E^ methods developed by Waters [63].

Because the precursors, primary, and internal fragments have different modulation behavior, they show different patterns in the vertical dimension. Figure 4 shows how phase shifts occur in the vertical dimension for the MH_3_^3+^ precursor ion, a primary fragment ion (*b*_9_^2+^), and a secondary fragment ion (IH^+^) of angiotensin 1. Figure 4a–c show the mapping of the fragmentation zone in IRMPD for the three ion species with the pulse sequence in Scheme 1b. Figure 4a shows the evolution of the intensity of MH_3_^3+^ with the amplitude of the first pulse. The intensity increases with the amplitude, which is consistent with the fact that IRMPD fragmentation happens at the center of the ICR cell where the photon density is highest. Figure 4a also shows that, at the center of the ICR cell, the fragmentation efficiency is close to 100%, since the intensity of the signal for MH_3_^3+^ is almost zero. Figure 4b shows the evolution of the intensity of fragment *b*_9_^2+^, which is a primary fragment ion. At high pulse amplitude (i.e., high precursor ion radius), the intensity of the fragment ion peak is low because the photon density encountered by the precursor ion packet is low. The intensity of *b*_9_^2+^ increases as the radius of the precursor ion packet decreases (i.e., the photon density increases), until the photon density is high enough that *b*_9_^2+^ undergoes secondary fragmentation, and the intensity decreases again. The fragmentation zone that produces *b*_9_^2+^ is therefore shaped like two concentric cylinders. Figure 4c shows the evolution of the intensity of IH^+^, which is an internal fragment of angiotensin 1, and most likely a product of secondary fragmentations of angiotensin 1. The intensity of the peak of IH^+^ is low at high radius and low photon density, and increases at low radius and high photon density, which is the opposite behavior to the intensity of the precursor ion in Figure 4a. Its fragmentation zone is cylindrical.

Figure 4d–f follow the evolution of the magnitude mode intensity of the peaks for the same ion species in the pulse sequence from Scheme 1a for increasing values of the encoding delay *t*_1_. Figure 4d shows the evolution of the intensity of the MH_3_^3+^ precursor with *t*_1_. The intensity alternates between high intensity (high ion packet radius during the fragmentation period) and low intensity (low ion packet radius during the fragmentation period). Because the excitation pulse in the pulse sequence excites ion packets at a radius that is approximately 25 times higher than the maximum radius after the encoding sequence, the radius modulation of the precursor ion alone is low. However, since the fragmentation efficiency of MH_3_^3+^ of angiotensin is almost 100% at the center of the ICR cell, its signal intensity modulation is at its maximum amplitude [17]. The modulation frequency for MH_3_^3+^ is approximately 300 kHz, which is consistent with a cyclotron frequency of 425 kHz and the lowest frequency in the frequency sweep being 122.8 kHz [2, 4, 17].

Figure 4e shows the modulation of the intensity of the *b*_9_^2+^ fragment, which has the same frequency as its precursor. Because of its ring-shaped fragmentation zone, the intensity shows sharp peaks when the precursor ion packet radius increases or decreases.

Figure 4f shows the modulation of the intensity of the IH^+^ fragment, which has a cylindrical fragmentation zone at the center of the ICR cell. Its intensity is modulated at the same frequency as the MH_3_^3+^ precursor and the *b*_9_^2+^ fragment, but it is phase-shifted by 180° compared with the MH_3_^3+^ precursor, as predicted.

The signal intensity modulation for both *b*_9_^2+^ and IH^+^ are shifted by 180° compared with the signal intensity modulation of MH_3_^3+^. This result can help increase the accuracy of the correlation between precursor and fragment ions. Another observation is that the signal intensity modulation has a different shape for *b*_9_^2+^ (primary fragment) and IH^+^ (secondary fragment). As a result, the harmonic peaks in the vertical precursor ion scans will have different relative intensities for the two fragments. The vertical precursor ion scan for *b*_9_^2+^ can be expected to have a higher relative intensity for harmonics than in the vertical precursor ion scan for IH^+^. The relative intensities of harmonic peaks have already been shown to contain useful information for peptide de novo sequencing with IR-ECD as a fragmentation mode [14]. The data presented here shows that harmonic peaks can also be used to improve the accuracy of peptide sequencing with IRMPD as a fragmentation mode by differentiating *a*,*b*/*y* fragments from internal fragments.

Figure 5a shows the normalized signal intensity modulation of different isotopes of MH_3_^3+^ of angiotensin 1 (^12^C, 1×^13^C, and 2×^13^C) as a function of *t*_1_. All isotopes have the same fragmentation behavior, but they have different cyclotron frequencies and modulation frequencies. The difference in modulation frequencies is 0.33 kHz between the ^12^C and 1×^13^C isotopes and 0.327 kHz between the 1×^13^C and 2×^13^C isotopes. According to Eq. (1), the radius modulation of precursor ion packets *r*(*t*_1_) can be expressed as:

24 $$ r\left({t}_1=0\right)={r}_0{\left(2\left(1+\cos {\omega}_1T\right)\right)}^{1/2} $$

in which *r*_0_ is the radius of the precursor ions at the end of the first pulse, *ω*_1_ is the cyclotron frequency of the precursor ions, and *T* is the length of the first pulse. The phase shift for a precursor ion can therefore be calculated as *ω*_1_*T.* Here, *T* = 0.7216 ms. The predicted phase difference between the ^12^C and 1×^13^C isotopes is therefore 13.6° and the predicted phase difference between the 1×^13^C and 2×^13^C isotopes is 13.5°. This phase shift can be clearly observed between the three isotopes in Figure 5a, where the signal intensity modulation is shifted along *t*_1_. The measured time shift is 0.6 μs, which corresponds to a phase shift of 15°, which is within the expected error for the experimental data.

The cyclotron frequency–dependent phase shift for the precursor ion signal intensity modulation and the correlation between precursor ion and fragment ion signal intensity modulation means that the phase information contained in the 2D MS data sets along *t*_1_ can be used to correlate precursor and fragment ions. Figure 5b–d show the shift between the ^12^C and 1×^13^C isotopes of the (HPF-H_2_O)^+^ fragment at *m/z* 364.1768, the *y*_9_^3+^ fragment at *m/z* 394.5575, and the *b*_9_^2+^ at *m/z* 583.2987 respectively. The same 15° phase shift as the MH_3_^3+^ precursor can be found between the signals of the isotopes of all three fragments.

Within a 360° phase cycle, the phase information in the vertical dimension can therefore be used to increase the accuracy of the correlation between precursor and fragment ion m/z ratios. In the present data set, with a pulse length of *T* = 0.7216 ms, the corresponding frequency range is 1.39 kHz, which corresponds to an *m/z* 1.2 range at *m/z* 400. Within an *m/z* 1.2 range in the precursor ion dimension, phase information can be used for fragment ion peaks to match them with precursors that cannot be resolved in the magnitude mode 2D mass spectrum, which is useful in the case of overlapping precursor ion isotopic distributions.

## Conclusion

In 2D mass spectrometry, the two consecutive Fourier transformations mean that there is a horizontal and a vertical phase contribution to the results. In the horizontal dimension, the phase function at the start of detection is different for precursor and fragment ions. The precursor ion phase function is *t*_1_-dependent. Furthermore, some ion species can be both precursor and fragment of another precursor (e.g., by neutral loss, or by electron capture), and therefore exist in two ion populations that exhibit different phase behaviors. The peaks corresponding to these ions are expected not to follow either the precursor or the fragment ion phase function, but rather a combination of both.

In the vertical precursor ion dimension, the signal intensity behavior is different for precursor and fragment ions, and it also depends on the fragmentation, which has a shape that depends on whether a fragment is the product of primary or secondary fragmentation. This result can be used to differentiate between primary and secondary fragments, for a better understanding of fragmentation patterns and kinetics, and to improve confidence in assignments. Precursor and fragment signal intensity modulations are shifted by 180°. The precursor ion signal intensity modulation has a phase that depends on their cyclotron frequency. Phase information in the vertical dimension can therefore be used to improve the accuracy of the correlation between precursor and fragment ion peaks.

In 2D MS, unlike in one-dimensional FT-ICR MS or Orbitrap MS, the phase information depends as much on chemistry as on physics. Any method that is developed to calculate a phase-corrected absorption-mode 2D mass spectrum can therefore be expected to involve compromise in the quality of the data. However, phase information in 2D mass spectra is a fruitful area of investigation and may lead to improved accuracy in the correlation between precursor and fragment ions without requiring longer experiments or increased sample consumption. Therefore, absorption mode in the vertical dimension is far more complex than the horizontal dimension, but nevertheless is informative.

## Electronic supplementary material

ESM 1 (PDF 653 kb)

## Abbreviations

2D MS: two-dimensional mass spectrometry
FT-ICR MS: Fourier transform ion cyclotron resonance mass spectrometry
IRMPD: infrared multiphoton dissociation
ECD: electron capture dissociation
SWIFT: stored waveform inverse Fourier transform
SWIM: stored waveform ion radius modulation
CAD: collisionally activated dissociation
NMR: nuclear magnetic resonance
nESI: nano electrospray
IR-ECD: infrared activated electron capture dissociation
