# Supplementary material for: Phase relationships in two-dimensional mass spectrometry
Source: J Am Soc Mass Spectrom. 2019 Oct 15;30(12):2594–607. doi: 10.1007/s13361-019-02308-1 (PMC6914722; doi:10.1007/s13361-019-02308-1)
Supplement: Supplementary file 1 — (PDF 653 kb) [file 13361_2019_2308_MOESM1_ESM.pdf]

Phase relationships in two-dimensional mass spectrometry

Journal of the American Society for Mass Spectrometry

Maria A. van Agthoven[§], *David P.A. Kilgour,*[§†] *Alice M. Lynch,*[§] *Mark P. Barrow,*[§] *Tomos Morgan,*[§] *Christopher A. Wootton,*[§] *Lionel Chiron,*[+] *Marc-André Delsuc,*[#+] *Peter B. O'Connor*[§*]

[§] Department of Chemistry, University of Warwick, Gibbet Hill Road, CV4 7AL Coventry, United Kingdom

[+] CASC4DE, Le Lodge, 20 av. du Neuhof, 67100 Strasbourg, France

[#] Institut de Génétique et de Biologie Moléculaire et Cellulaire, INSERM, U596; CNRS, UMR7104; Université de Strasbourg, 1 rue Laurent Fries, 67404 Illkirch-Graffenstaden, France

Present Addresses

[†]School of Science and Technology, Nottingham Trent University, 50 Shakespeare Street, NG1 4FQ Nottingham, United Kingdom.

[%]Department of Computer Science, University of St Andrews, North Haugh, St Andrews, Fife, KY16 9SX, United Kingdom.

Corresponding author:

Address reprint requests to:

*Peter O'Connor, Department of Chemistry, University of Warwick, Gibbet Hill Road, CV4 7AL Coventry, United Kingdom

e-mail: p.oconnor@warwick.ac.uk

telephone: +44 (0)2476 151008

Supporting Information

Contents:

Figure S1. ECD MS/MS spectrum of substance P in absorption mode.

Figure S2. Absorption mode ECD tandem mass spectra for substance P using the pulse sequence in Scheme 1a with a t1 delay of 1.0-5.0 µs The column on in the left middle shows the peak assigned to the MH22+ precursor. The two right columns on the left and right show the peak for the c5 and c7 fragments for different values of t1 respectively. The mass spectra have not been internally calibrated.

Figure S3. Absorption mode ECD tandem mass spectra for substance P using the pulse sequence in Scheme 1a with a t_1 delay of 1.0-5.0 µs The column on in the left middle shows the peak assigned to the MH_2^{2+} precursor. The two right columns on the left and right show the peak for the c_5 and c_7 fragments for different values of t_1 respectively. The mass spectra have not been internally calibrated. All five spectra were phased with the same phase coefficients.

Table S1. Peak assignments for spectrum in Fig. S1.

Table S2. Peak assignments for spectrum in Fig. 1 in magnitude mode.

Table S3. Peak assignments for the spectra of angiotensin 1 in Fig. 3a in magnitude mode.

Table S4. Peak assignments for the spectra of angiotensin 1 in Fig. 3b in magnitude mode.

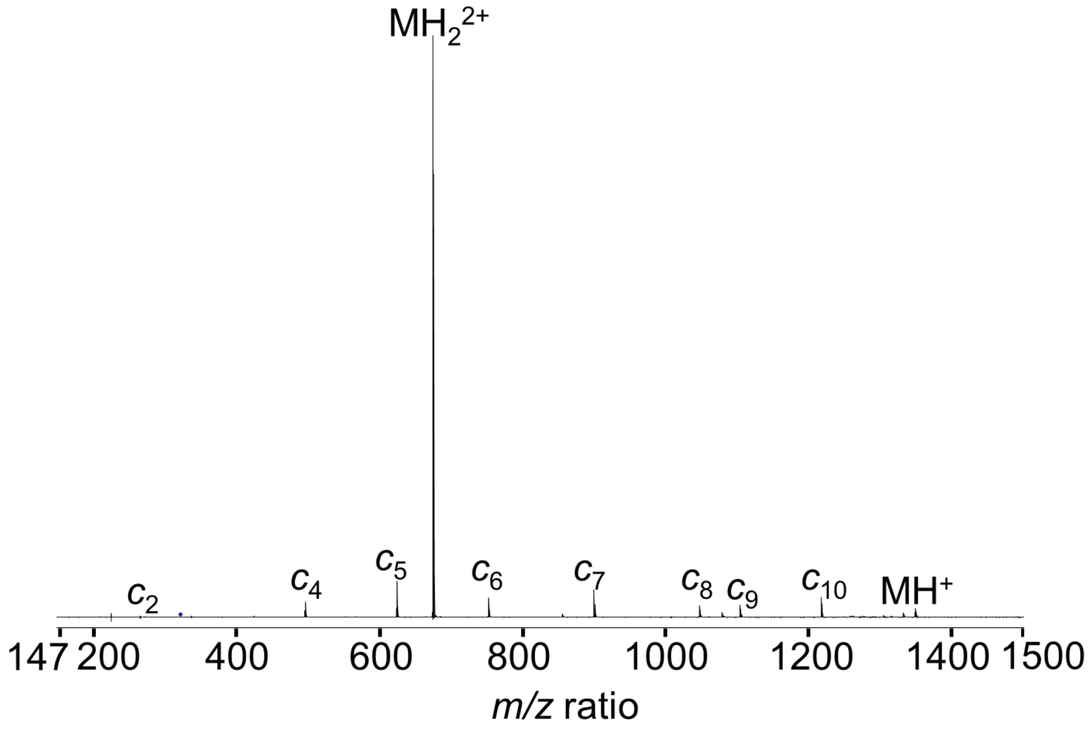

Figure S1. ECD MS/MS spectrum of substance P in absorption mode.

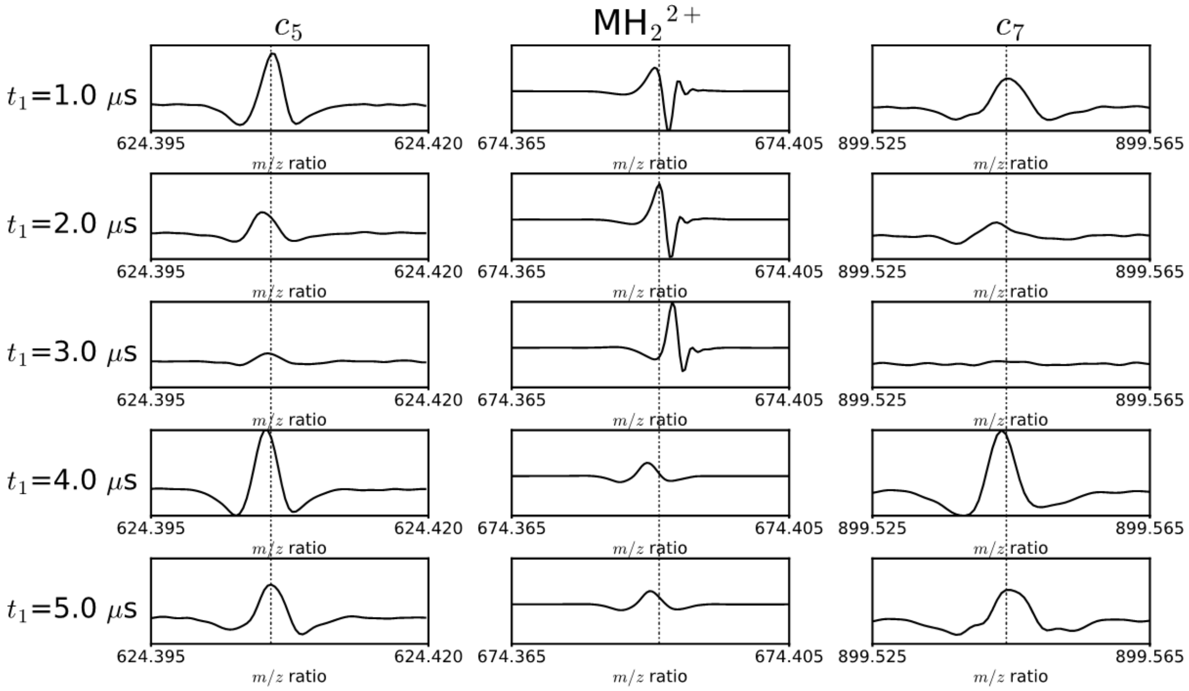

Figure S2. Absorption mode ECD tandem mass spectra for substance P using the pulse sequence in Scheme 1a with a t_1 delay of 1.0-5.0 µs The column on in the left middle shows the peak assigned to the MH_2^{2+} precursor. The two right columns on the left and right show the peak for the c_5 and c_7 fragments for different values of t_1 respectively. The mass spectra have not been internally calibrated.

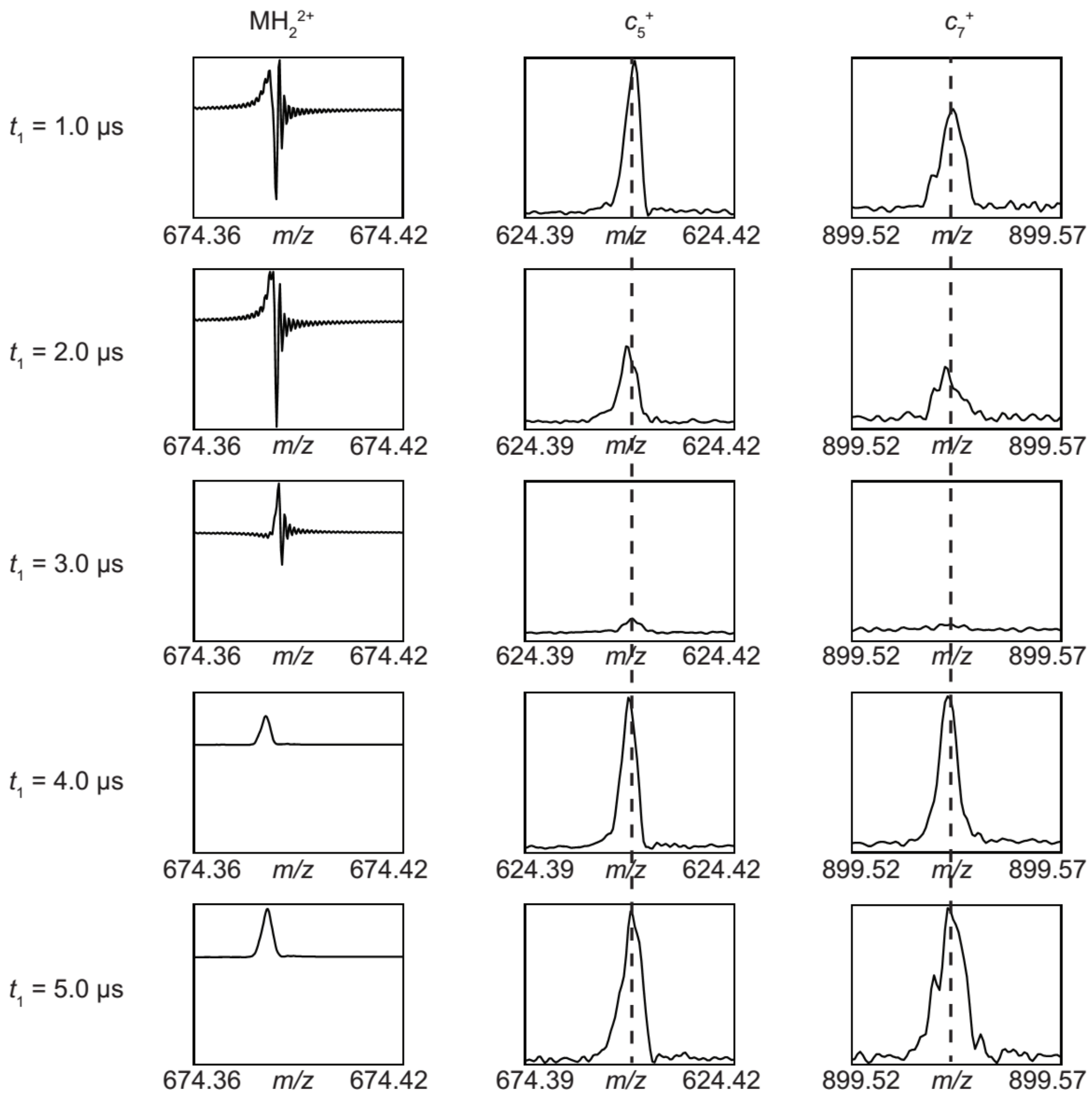

Figure S3. Absorption mode ECD tandem mass spectra for substance P using the pulse sequence in Scheme 1a with a t_1 delay of 1.0-5.0 µs The column on in the left middle shows the peak assigned to the MH_2^{2+} precursor. The two right columns on the left and right show the peak for the c_5 and c_7 fragments for different values of t_1 respectively. The mass spectra have not been internally calibrated. All five spectra were phased with the same phase coefficients, which were calculated with the mass spectrum shown in Fig. S1.

Table S1. Peak assignments for spectrum in Fig. S1.

m/z ratio	Peak assignment	Mass accuracy (ppm)	S/N ratio*
271.18770	c_2	0.00	6
495.32757	$c_4^{\bullet}$	-0.06	69
496.33550	c_4	0.15	170
623.38619	$c_5^{\bullet}$	0.02	91
624.39407	c_5	0.10	360
674.37104	MH_2^{2+}	-0.46	5700
751.44418	$c_6^{\bullet}$	-0.77	13

752.45265	c_6	0.09	180
898.51361	$c_7^{\bullet}$	0.49	5
899.52097	c_7	-0.03	240
1046.58948	c_8	0.07	94
1078.56283	z_9	0.01	42
1103.61111	c_9	0.21	93
1216.69523	c_{10}	0.24	140
1347.73544	MH^+	0.01	30

Average of the absolute value of the mass accuracies: 0.18 ppm

*As calculated by the Data Analysis 4.0 software.

Table S2. Peak assignments for spectrum in Fig. 1 in magnitude mode.

m/z ratio	Peak assignment	Mass accuracy (ppm)	S/N ratio*
157.10852	b_1	0.85	9
254.16116	b_2	0.04	52
271.18771	c_2	0.04	59
449.91638	MH_3^{3+}	-0.62	770
496.3352	c_4	-0.46	90
547.79459	y_9^{2+}	0.32	45
552.30882	c_9^{2+}	-0.46	73
600.33761	b_{10}^{2+}	-0.37	59
608.85140	c_{10}^{2+}	0.48	41
624.39426	c_5	0.41	165
674.37376	MH_2^{2+}	3.6	9500
752.45294	c_6	0.48	72
899.51998	c_7	-1.1	38
1046.59056	c_8	1.1	15
1079.56806	$z_9^{\bullet}$	-2.4	8
1103.6105	c_9	-0.34	18
1216.69969	c_{10}	3.9	9
1347.73510	MH^+	-0.24	22

Average of the absolute value of the mass accuracies: 0.96 ppm

*As calculated by the Data Analysis 4.0 software.

Table S3. Peak assignments for the spectra of angiotensin 1 in Fig. 3a in magnitude mode.

**Primary fragments**				
m/z ratio	Relative intensity (% to the maximum of assigned fragment peaks)	Peak assignment	Mass accuracy (ppm)	S/N ratio*
254.12486	3.6	b_2-H_2O	0.37	24
255.10887	9.5	b_2-NH_3	0.34	67
269.16083	2.6	y_2	0.05	16
272.13534	9.4	b_2	0.03	65
353.19295	2.2	b_3-H_2O	-0.65	13
354.17698	2.2	b_3-NH_3	-0.61	13
383.70309	1.9	$(b_6\text{-}H_2O)^{+2}$	-0.76	10
392.70837	4.0	b_6^{+2}	-0.75	25
513.28166	3.7	y_4	-0.65	21
514.76881	1.5	b_8^{+2}	-0.86	7
516.25619	2.6	b_4-H_2O	-0.62	14
517.24023	2.4	b_4-NH_3	-0.57	13
534.26680	6.7	b_4	-0.51	39
583.29752	2.5	b_9^{+2}	-2.03	13
602.32960	2.1	a_5-NH_3	-0.12	10
619.35637	8.4	a_5	0.24	47
629.34069	3.1	b_5-H_2O	0.19	17
630.32464	4.6	b_5-NH_3	0.08	25
647.35144	18.1	b_5	0.47	104
650.34099	2.1	y_5	0.13	11
766.40073	4.6	b_6-H_2O	1.63	23
767.38479	4.2	b_6-NH_3	1.68	21
784.41156	11.4	b_6	1.93	59
1010.52515	2.9	b_8-H_2O	4.44	12
1028.52950	4.0	b_8	-1.68	17
**Secondary fragments**				
m/z ratio	Relative intensity (to the maximum of assigned fragment peaks)	Peak assignment	Mass accuracy (ppm)	S/N ratio*
217.13392	8.8	PF-CO	1.75	64
223.15566	3.5	IH-CO	1.44	24
228.18222	2.1	RV-CO	1.46	13
235.11921	17.4	HP	1.10	130
235.14437	5.9	VY-CO	1.13	41
239.15049	15.6	RV-NH_3	1.00	110
245.12865	4.2	PF	0.80	29
249.15988	5.1	YI-CO	0.51	35
251.15040	27.8	IH	0.59	200
256.17693	4.9	RV	0.50	34
257.13986	1.6	FH-CO	0.67	10
263.13906	11.5	VY	0.16	81

277.15471	2.0	YI	0.15	12
285.13455	8.4	FH	-0.18	57
364.17653	100.0	HPF-H_2O	-0.74	670
376.22278	1.9	VYI	-0.81	11
382.18706	19.3	HPF or PFH	-0.80	130
391.24491	8.5	RVY-CO	-0.78	54
402.21323	7.4	RVY-NH_3	-0.87	47
414.21327	3.4	YIH	-0.75	21
419.23977	21.4	RVY	-0.86	140
495.27111	11.8	IHPF	-0.65	73
504.32894	46.3	RVYI-CO	-0.67	290
515.29736	3.6	RVYI-NH_3	-0.55	20
532.32399	28.5	RVYI	-0.38	170
641.38831	4.6	RVYIH-CO	0.19	260
652.35674	9.3	RVYIH-NH_3	0.28	530
669.38349	17.2	RVYIH	0.57	97
757.40440	2.2	VYIHPF	1.62	10
885.51145	1.7	RVYIHPF-CO	2.35	6
896.48062	3.4	RVYIHPF-NH_3	3.22	15
913.50703	7.8	RVYIHPF	3.01	37

Average of the absolute value of the mass accuracies: 0.90 ppm

*As calculated by the Data Analysis 4.0 software.

Table S4. Peak assignments for the spectra of angiotensin 1 in Fig. 3b in magnitude mode.

**Primary fragments**				
m/z ratio	Relative intensity (% to the maximum of assigned fragment peaks)	Peak assignment	Mass accuracy (ppm)	S/N ratio*
257.14464	5.0	y_4^{+2}	0.02	79
269.16083	2.8	y_2	0.05	42
272.13537	0.5	b_2	0.14	6
310.18159	0.5	a_5^{+2}	-0.51	7
325.67404	2.0	y_5^{+2}	-0.16	29
370.19725	1.5	$(a_6\text{-NH}_3)^{+2}$	-1.84	20
378.71120	8.5	a_6^{+2}	-0.01	130
379.86992	2.2	a_9^{+3}	-0.02	32
383.70333	5.3	$(b_6\text{-H}_2\text{O})^{+2}$	-0.13	78
384.19518	1.0	$(b_6\text{-NH}_3)^{+2}$	-0.54	13
389.20159	5.1	b_9^{+3}	0.07	76
392.70865	45.2	b_6^{+2}	-0.03	680
394.55743	62.1	y_9^{+3}	-0.07	930
395.20499	4.6	$(b_9\text{+H}_2\text{O})^{+3}$	-0.24	67
416.22844	0.7	y_3	-1.90	8
426.89621	8.8	$(\text{MH}_3\text{-H}_2\text{O})^{+3}$	-0.10	130
427.22423	1.4	$(\text{MH}_3\text{-NH}_3)^{+3}$	-0.06	19
500.77161	0.8	a_8^{+2}	-0.37	10
505.76387	0.6	$(b_8\text{-H}_2\text{O})^{+2}$	-0.20	6
513.28193	25.6	y_4	-0.13	360
514.76907	3.4	b_8^{+2}	-0.35	46
534.26708	1.5	b_4	0.01	20
569.30114	3.8	a_9^{+2}	-0.19	51
574.29349	4.8	$(b_9\text{-H}_2\text{O})^{+2}$	0.11	63
574.78544	2.3	$(b_9\text{-NH}_3)^{+2}$	0.01	29
583.29864	100.0	b_9^{+2}	-0.11	1400
592.30397	26.7	$(b_9\text{+H}_2\text{O})^{+2}$	-0.03	360
647.35115	9.0	b_5	0.02	120
766.39954	1.1	$b_6\text{-H}_2\text{O}$	0.07	11
784.41015	8.0	b_6	0.13	94
1028.53108	1.2	b_8	-0.14	11
**Secondary fragments**				
m/z ratio	Relative intensity (to the maximum of assigned fragment peaks)	Peak assignment	Mass accuracy (ppm)	S/N ratio*
235.11896	1.1	HP	0.03	16
251.15024	2.1	IH	-0.05	32
354.19369	1.3	PFH-CO	3.50	18
364.1767	4.2	HPF-H_2O	-0.22	62
382.18728	4.5	HPF	-0.23	65
419.24013	0.9	RVY	0.00	11

495.27138	8.8	IHPF	-0.10	120
504.32939	1.4	RVYI-CO	0.22	18
519.24621	0.8	HPFH	-0.13	10
532.32411	6.4	RVYI	-0.16	87
669.38320	3.7	RVYIH	0.14	47

Average of the absolute value of the mass accuracies: 0.32 ppm

*As calculated by the Data Analysis 4.0 software.
